# Roles of Dynein and Dynactin in Early Endosome Dynamics Revealed Using Automated Tracking and Global Analysis

**DOI:** 10.1371/journal.pone.0024479

**Published:** 2011-09-06

**Authors:** Neftali Flores-Rodriguez, Salman S. Rogers, David A. Kenwright, Thomas A. Waigh, Philip G. Woodman, Victoria J. Allan

**Affiliations:** 1 Faculty of Life Sciences, University of Manchester, Manchester, United Kingdom; 2 School of Physics and Astronomy, University of Manchester, Manchester, United Kingdom; 3 Photon Science Institute, University of Manchester, Manchester, United Kingdom; Institut Européen de Chimie et Biologie, France

## Abstract

Microtubule-dependent movement is crucial for the spatial organization of endosomes in most eukaryotes, but as yet there has been no systematic analysis of how a particular microtubule motor contributes to early endosome dynamics. Here we tracked early endosomes labeled with GFP-Rab5 on the nanometer scale, and combined this with global, first passage probability (FPP) analysis to provide an unbiased description of how the minus-end microtubule motor, cytoplasmic dynein, supports endosome motility. Dynein contributes to short-range endosome movement, but in particular drives 85–98% of long, inward translocations. For these, it requires an intact dynactin complex to allow membrane-bound p150*^Glued^* to activate dynein, since p50 over-expression, which disrupts the dynactin complex, inhibits inward movement even though dynein and p150*^Glued^* remain membrane-bound. Long dynein-dependent movements occur via bursts at up to ∼8 µms^−1^ that are linked by changes in rate or pauses. These peak speeds during rapid inward endosome movement are still seen when cellular dynein levels are 50-fold reduced by RNAi knock-down of dynein heavy chain, while the number of movements is reduced 5-fold. Altogether, these findings identify how dynein helps define the dynamics of early endosomes.

## Introduction

The motility of organelles within the endocytic pathway contributes to the passage and sorting of endocytosed material [1], [2], [3], as well to the spatial organization of endosomal signalling platforms [4], [5]. This motility is powered by the activity of motor proteins that drive endosome movement along microtubules and/or actin [6]. Early endosomes interact with many motor proteins, including the minus-end microtubule motor dynein [7], [8], [9], [10], [11], [12], [13], [14], minus-end kinesins such as KIFC1 [15] and KIFC2 [16], and plus-end kinesins including kinesin-1 [8], [15], [17] and kinesin-3 family members such as KIF16B [18] and others [10], [19]. In addition, myosins [20], [21], proteins that regulate binding of endosomes to actin [22], [23], and actin polymerization [3] also control early endosome dynamics.

Despite the identification of such a wide range of effectors controlling endosome motility, there has been no systematic description of the complex endosome movement that is observed in vivo, and how particular motor proteins and cytoskeletal regulators contribute to each component of this behaviour [7], [9],[10],[11],[12],[24],[25],[26],[27],[28]. Addressing this requires precise evaluation of the properties of early endosome motion, both in global terms and at the level of individual endosomes, together with an identification of how specific molecular motor(s) contribute to this motion. Here, we have used automated particle tracking [29] of early endosomes imaged at high frame rates, and applied a recently developed computational algorithm [30] to provide accurate and quantitative information about early endosome movement. This global analysis shows how dynein contributes to overall early endosome motion, supporting many short-range movements and nearly all fast, long-range inward movements. In addition, by examining the movement of individual endosomes under different conditions, we show that peak speeds are surprisingly insensitive to motor copy number.

## Results and Discussion

### Imaging and tracking early endosomes at high temporal and spatial resolution

We analyzed the movement of early endosomes, defined by the presence of Rab5, by imaging at 28 frames s^−1^. To label them without perturbing their function, GFP-Rab5 was expressed in HeLaM cells at 10–20% of endogenous Rab5 levels (Figure S1A). GFP-Rab5 endosomes colocalized extensively with the early endosomal markers EEA1 and Hrs, and with briefly internalized EGF, but not with the lysosomal marker LAMP1 (Figure S2). They were highly motile (Movie S1), with many moving rapidly towards or away from the cell centre, and occasionally reversing direction. Some exhibited short-range motility or moved very little, whilst others alternated between this behaviour and long-range movement. Similar motility was seen in RPE cells, which have a more radial microtubule network than HeLaM cells (Movie S2).

**Figure 1:**
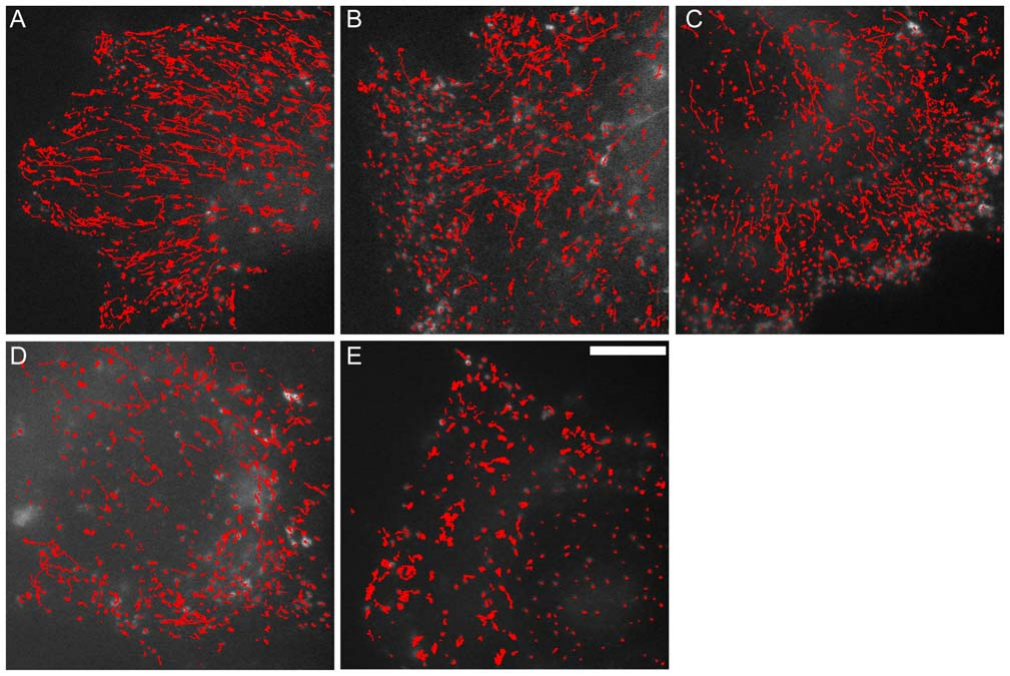
PolyParticleTracker tracks early endosome movement accurately. Movies of GFP-Rab5 were recorded for 1000 frames at 28 frames s^−1^ in HeLaM cells: (A) control cell; (B) DHC1 depleted cell; (C) p50 expressing cell; (D) CC1 expressing cell; (E) nocodazole-treated cell. PolyParticleTracker was applied to each movie, and the resulting tracks overlaid on the first movie frame (bar = 10 µm).

To obtain spatial information about this complex behaviour on the nanometer scale and accurate estimates of endosome speed, we exploited PolyParticleTracker, a method recently developed by the authors Salman Rogers and Tom Waigh, with Xiubo Zhao and Jian Lu in the Biological Physics group, University of Manchester [29]. Approximately 1,800 GFP-Rab5 endosomes were tracked with high fidelity using PolyParticleTracker during each 1,000 frame movie (Figure 1A). *Runs* were defined as movements of ≥85 nm, with a rate of ≥0.17 µms^−1^, while movements of <85 nm in 0.5 s were defined as *rests* [30]. On average, endosomes in HeLaM cells spent 22% of total time running, with 65% of endosomes exhibiting at least one *run* during the recording (Table S1). The proportion of endosomes that moved was lower in RPE than HeLaM cells (39%), and therefore a lower percentage of total track time was spent running (Table S1). However, those endosomes that moved covered long distances in both cell types (Figure 1A and Figure S3A). Endosome movement occurs along microtubules, since essentially no linear movement was observed in HeLaM cells treated with nocodazole to depolymerize microtubules (Figure 1E and Table S1).

### Dynein drives both short and long range early endosome motility

We have previously identified dynein as the motor that drives the inward movement of EGF-containing early endosomes in HeLa cells, but this analysis was performed at low temporal resolution (1 frame s^−1^: [7]). To assess how dynein contributes to the movement of all GFP-Rab5 endosomes, HeLaM cells were imaged at 28 frames s^−1^ under three conditions that disrupt dynein function. Firstly, we over-expressed the dynactin subunit, p50, which disrupts dynactin complex integrity [31]. Dynactin is a large protein complex that interacts with dynein and is required for dynein function in vivo. Secondly, cells were transfected with a peptide, corresponding to the N-terminal coiled coil region (CC1) of the dynactin p150 subunit, which prevents dynein-dynactin binding [31]. Thirdly, cells were depleted of the heavy chain of cytoplasmic dynein 1 (DYNC1H1, referred to as DHC1 herein) by ∼98% using siRNA (Figure S1B-D). These interventions greatly reduced GFP-Rab5 movement (Movie S1, Figure 1B-D and Table S1), and increased the proportion of stationary endosomes (Table S1), confirming that dynein is vital for early endosome motility [7], [8], [9], [10], [11], [12], [13]. GFP-Rab5 endosome movement in RPE cells was also profoundly inhibited by DHC1 depletion, or by p50 expression (Movie S2, Figure S1C, Figure S3A-C and Table S1).

**Figure 2:**
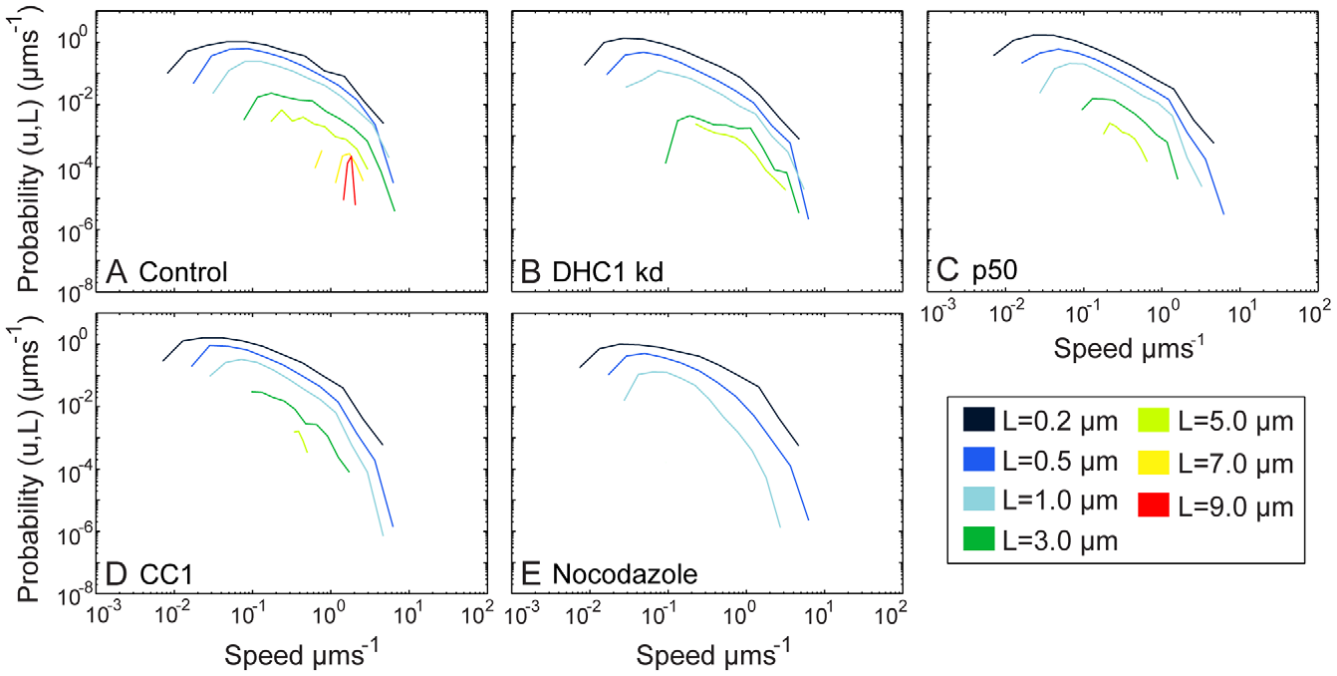
First Passage Probability measurements allow a global description of early endosome movement. Tracking data from 5 movies in HeLaM cells for each condition were used for FPP analysis of GFP-Rab5 in: (A) control cells; (B) DHC1 depleted cells; (C) p50 expressing cells; (D) CC1 expressing cells; (E) nocodazole-treated cells. For each length of passage, *L* (see key), the probability of that passage occurring at a given average speed is plotted.

To provide an unbiased, systematic description of endosome motility, we applied First Passage Probability (FPP) analysis, a method used to measure many fundamental stochastic processes [32]. We have shown that FPP reveals the behaviour of the endosome population in terms of distances moved, and average speeds over these distances [30]. It does this by measuring the likelihood that an endosome will require a time *t* before it reaches a displacement *L* (or *passage*) along a track, for the first time [30]. These measurements are converted into average speeds and plotted for different values of *L* (Figure 2, HeLaM cells and Figure S3, RPE cells). Our studies of endosome movement at moderate temporal resolution show that FPP provides a reproducible, non-biased analysis of particle motion [30]. Importantly, in contrast to ensemble measurements such as mean-squared displacement analysis, FPP allows different patterns of behaviour within a population to be distinguished, and therefore readily highlights the small percentage of endosomes that move rapidly over long distances. We therefore applied FPP analysis to endosome movement in cells with and without functional dynein.

**Figure 3:**
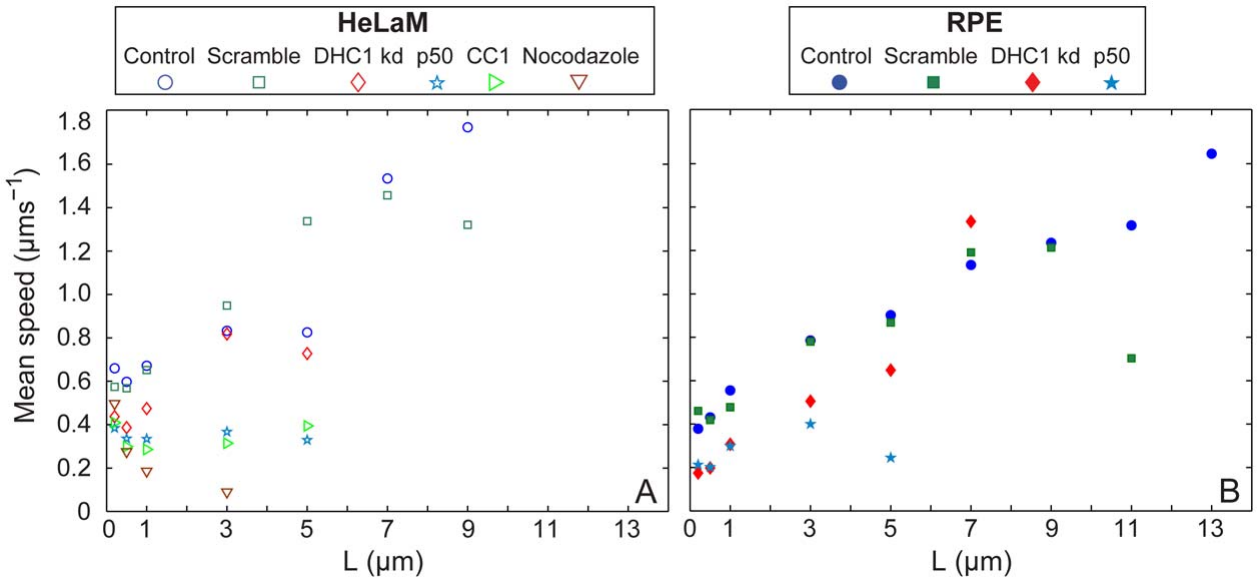
FPP analysis identifies distinct short and long-range endosome dynamics. FPP measurements of endosome movement for each condition (see key) in HeLaM cells (A), or RPE cells (B), were plotted to show the mean passage speed for given passage lengths.

FPP analysis of control cells revealed that a small proportion of endosomes underwent long passages of up to 9 µm in HeLaM cells (Figure 2A) or 13 µm in RPE cells (Figure S3D). Endosomes travelled further in RPE cells than in HeLaM cells, possibly because of their more radial microtubule network, or simply because the distance between the cell periphery and the nucleus is longer in the larger RPE cells. There are many more short passages than long ones, because most endosomes move only short distances at any given time, as seen in movie S1 and movie S2 (although each long passage will of course contain multiple short passages, accounting for some of the total short passages that are scored). In control cells the distribution of speeds was very broad for short passages, but narrowed considerably as passage length increased, with the longest passages being achieved at speeds of 1–2 µms^−1^ (Figure 2A and Figure S3D). In both cell types the mean speed increased with run length (Figure 3A–B), and notably, speeds of up to 6 µms^−1^ were seen for 3.0 µm passages (Figure 2A and Figure S3D).

We next applied FPP analysis to identify how inhibiting dynein function affected microtubule-based endosome motility. First, to provide a baseline, we analysed nocodazole-treated HeLaM cells. Very few movements longer than 1.0 µm were seen in the absence of microtubules (Figure 2E and Figure 3A), and the mean speed 0.5, 1.0 and 3.0 µm passages was greatly reduced compared to control cells (Figure 3A). However, a small proportion of 0.5 µm passages still reached 6 µms^−1^, revealing a population of microtubule-independent rapid, short-range movements (Figure 2E). These could be actin-based, or diffusional.

Disrupting dynein-dynactin interactions by expression of chicken p50 or human CC1 caused a complete loss of passages ≥7.0 µm and a dramatic reduction in the likelihood of 5.0 µm passages occurring in HeLaMs (Figure 2C–D). p50 expression caused a similar inhibition in RPE cells (Figure S3F). The mean speeds no longer increased with passage length, and were greatly reduced compared to controls in both cell types (Figure 3), as were the maximum passage speeds (Figure 2 and Figure 3). Depletion of DHC1 in HeLaM cells also led to the loss of ≥7.0 µm passages, and 5.0 µm passages were reduced 2-fold compared to control cells (Figure 2B). In DHC1-depleted RPE cells, both 5.0 and 7.0 µm passages still persisted, but with a greatly decreased probability (Figure S3E). However, no passages of ≥9.0 were seen. Importantly, the reduced number of endosomes that moved in DHC1-depleted cells did so with similar or only slightly reduced rates compared to controls in both cell types (Figure 2, Figure S3 and Figure 3). Altogether, these data are consistent with dynein driving long range endosome movements, and suggest that the small amount of dynein present after DHC1-depletion can drive fairly normal movement of a few endosomes. Furthermore, they suggest that dynein may also play a role in driving a relatively fast subset of short-range movements.

FPP analysis therefore provides a global description of endosome dynamics that highlights different aspects of their motility and shows the importance of dynein to each component. These data support a model in which endosomes switch in a probabilistic fashion between periods of motion (i.e. *runs*) and stationary pauses (*rests*) [30]. They mainly move only short distances, but occasionally undertake longer, faster excursions. The data are also consistent with the possibility that endosomes undergoing long *runs* can switch between faster and slower speeds. These dynamics may be intrinsically advantageous for combining the endosome functions of transporting endocytosed receptors and organizing signalling networks. It may be that long translocations are actively paused or terminated, as previously proposed for lipid droplets [33]. This would ensure that an endosome changes from long to short range motion or halts multiple times during its progress from the cell periphery towards centrally located lysosomes [1]. Interludes of shorter-range motion may be crucial for endosome functions such as sorting [2], [7], [34], where dynein may contribute to the organization and/or separation of endosomal domains. Dynein drives the vast majority of long range passages in both HeLaM and RPE cells, which would be essential for endosomal trafficking [7], [35] and signalling over distance [36]. Hence, although other motors may support long range early endosome movement [15], [16], these are most likely linked to a minor population of endosomes or function in specialized cells.

### Long range endosome movement occurs in short, very rapid bursts

**Figure 4:**
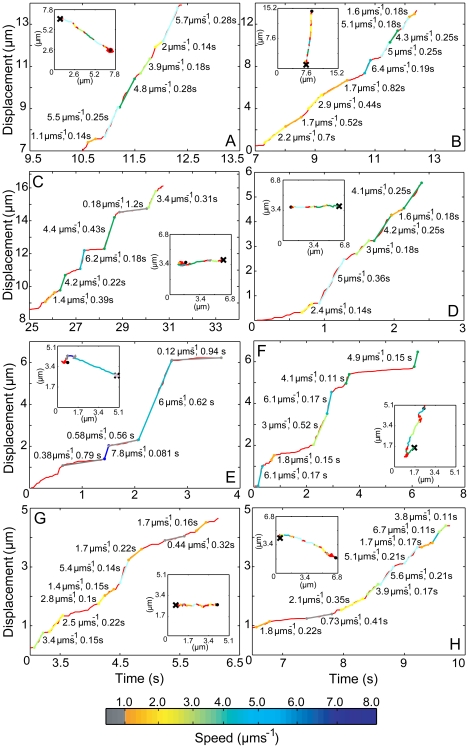
Early endosomes move in fast bursts, irrespective of cellular dynein levels. Representative tracks >2 µm in control RPE cells (A–B), DHC1-depleted RPE cells (C–D), control HeLaM cells (E–F), and DHC1-depleted HeLaM cells (G–H) were smoothed and plotted as displacement versus time (red lines). Constant speed segments were identified manually, colored according to the speed chart, and overlaid over the raw tracks (insets). The beginning (spots) and end (crosses) of each track is highlighted (inset), and the speed and duration of each segment is shown.

What are the features of such long range movements? Our high resolution tracking allowed us to examine these in greater detail. We selected inward tracks with a net displacement of >2 µm (Figure S4), since these should be largely if not exclusively dynein-dependent, based on their direction and our FPP analysis. Parsing analysis (see supporting methods) showed that in both RPE and HeLaM cells, each endosome track consisted of a series of constant speed segments of up to ∼8 µms^−1^ (Figure 4A–B and Figure 4E–F), greater than previous maximum speed estimates for endosomes in mammalian cells of 4–5 µms^−1^ found using lower temporal resolution imaging [25], [27], but in keeping with the FPP analysis of all particle tracks (Figure 2 and Figure S3). These rates are significantly higher than those observed in vivo for lysosomes [37], another organelle that uses dynein to move. Constant speed segments are also seen for purified vertebrate dynein in vitro [38], [39], but are ∼5–10 times slower than those observed here.

Endosome speed could change abruptly during a rapid translocation (Figure 4). In addition, bursts of movement were interrupted by slower translocations, or periods where endosomes were essentially static. Single endosomes usually moved at multiple rates within one track (Figure 4F). Strikingly, the distance moved during each constant speed segment remained fairly similar, irrespective of the speed of translocation (Table S2). In other words, as the average speed increased, the duration of movement became shorter such that even the fastest segments were limited to ∼1.4 µm on average (Table S2). Changes to dynein function may therefore be linked to the distance endosomes move. Alternatively, this distance may reflect the average spacing between obstacles that moving endosomes encounter.

Fast lateral or longitudinal translocation of microtubules can account for rapid membrane translocation under some circumstances [40]. To exclude microtubule sliding as a cause of the very fast endosome movements observed here, cells were imaged using mApple-Rab5 and a construct encoding the microtubule binding domain of ensconsin/E-MAP-115 tagged with three concatenated GFPs [41], [42], to visualize cargo and microtubules simultaneously (Movie S3). Whilst some microtubules were seen to move during the course of each movie, this movement was slow compared to that of the fastest moving endosomes. Moreover, of 185 long-distance endosome runs of >5 µms^−1^, identified at random from 20 movies, 112 were along microtubules that were essentially static (Movie S3 and Figure S5), 18 were on microtubules that displayed slow lateral or longitudinal movements, and only 2 occurred on fast moving microtubules (microtubules could not be tracked reliably alongside the remaining 43 endosome movements). Hence, most if not all, fast endosome movements are driven by endosome-associated dynein.

### Fast dynein-driven movement is sensitive to dynactin function but not reduction of cellular dynein levels

**Figure 5:**
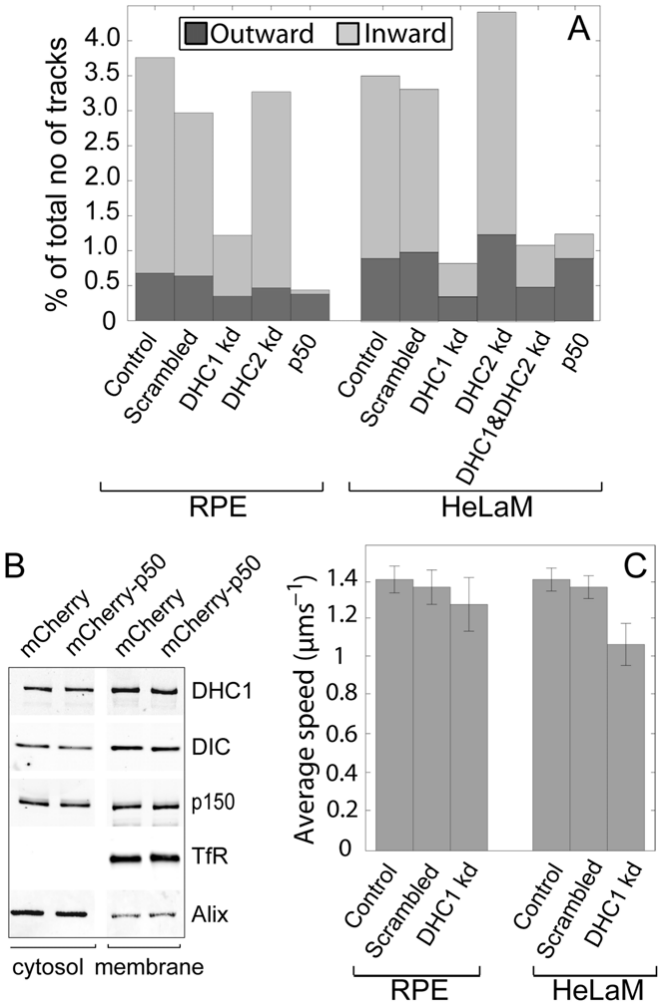
Effects of dynactin inhibition and DHC1 depletion on long-range endosome motility. (A) Histogram showing the % of total tracks that are >2 µm under each condition, in either direction. (B) Membrane association of dynein and dynactin components in extracts from transfected cells. TfR (transferrin receptor) is a membrane control and Alix is largely cytosolic. Note that membrane: cytosol loading is ∼4∶1. (C) Histogram showing average speeds of >2 µm tracks.

We next exploited our tracking experiments to address the importance of the presence of dynactin, and of cellular dynein levels, on cargo motility. In RPE cells expressing p50, long inward tracks were essentially abolished, and in HeLaM cells they were reduced by ∼85% (Figure 5A). Biochemical analysis revealed that dynein was not lost from membranes upon overexpression of p50 (Figure 5B). This is consistent with previous observations that the dynactin complex is not needed for dynein's membrane association in Drosophila [43], and under some conditions in *Neurospora crassa* [44], but contrasts with recent results from *Aspergillus nidulans* early endosomes [11]. Unexpectedly, the p150 subunit of dynactin also remained membrane associated upon p50 over-expression (Figure 5B). Therefore, the severe compromise in dynein activity is most likely because p150-dynein interactions can only activate dynein movement when p150 is part of an intact dynactin complex, as has been suggested recently for budding yeast dynein/dynactin [45]. Taken together, these data underscore the vital role played by dynactin in dynein function in vivo.

The influence of dynein copy number on cargo transport in vivo is a particularly controversial issue: one model proposes that engagement of more motors leads to faster movement [46], [47], whereas another suggests that while additional motors increase force production and distance moved, they may actually reduce speed [48], [49]. Here, DHC1 knock-down allowed us to test how cellular dynein levels influence the movement of a native cargo in living cells. DHC1 was depleted by ∼50-fold (Figure S1B-C), and biochemical analysis revealed that it was lost from the membrane fraction to the same extent as from soluble fractions (Figure S1D). While early endosomes will only be a minor component of the total membrane fraction, it is highly likely that their level of associated dynein is reduced in proportion to the general cellular depletion. The loss of DHC1 following depletion led to a fall in the number of moving endosomes (Movie S1 and Movie S2). The probability of long, inward tracks occurring fell by 75–80%, whilst long outward tracks were affected to a lesser extent (Figure 5A). Control movies using EB3-GFP to image the growing plus ends of microtubules [50], and immunofluorescence of fixed cells, showed that microtubule polarity and microtubule or actin organization were not affected by DHC1 depletion. Depletion of the heavy chain of cytoplasmic dynein 2 (DYNC2H1, referred to herein as DHC2), either alone or in combination with DHC1, did not affect the likelihood of long, inward tracks occurring (Figure 5A). This fits with cytoplasmic dynein 2 having a role in cilia assembly but not membrane movement [51].

A number of recent studies have provided estimates of total dynein number per membranous cargo. Biochemical approaches have revealed 1–5 molecules per mouse neuronal transport vesicle [37], while single molecule fluorescence studies have demonstrated that a single dynein per early endosome in *Ustilago maydis* is sufficient to drive their movement [28]. It may be more common to use a small number of motors, however, since force measurement suggest that there may be ∼5 active dyneins per *Dictyostelium* endosome [52]. Since we find that a 50-fold biochemical decrease in both membrane-associated and soluble DHC1 levels led to only a 4–5 fold decrease in long inward movements, those endosomes that still moved in DHC1-depleted cells would likely be left with a greatly reduced number of dynein molecules. Despite this, long, inward movements were often fast (Movie S1 and Movie S2), and were identified as such by FPP analysis (Figure 2, Figure 3 and Figure S3). In DHC1-depleted RPE cells, the average speed of endosomes during these long movements was virtually identical to that in control cells (Figure 5C), and this was also seen using FPP analysis (Figure 3B). Parsing of tracks again revealed the presence of multiple constant speed segments within one track (Figure 4C–D). Plotting the rates of constant speed segments as a cumulative frequency showed that their rates were only marginally altered in DHC1-depleted RPE cells compared to controls (Figure S6). Given that 50-fold less dynein is present following knock-down, these data suggest that changes in rates are not due to engagement or disengagement of extra dyneins. Endosomes in HeLaM cells depleted of DHC1 behaved similarly, though there was some reduction in average speeds (Figure 4G–H, Figure 5C and Figure S6).

Our data, in combination with those from other studies [28], [37], [52], reveal that low numbers of dyneins can move a cargo at rapid speeds in vivo, and contradict a previous study proposing a linear relationship between cargo speed and dynein copy number [46]. It is therefore unlikely that the disparity between endosome speeds in vivo and dynein motility in vitro can be explained simply by membrane-associated dynein acting as a multiple-motor unit. Fast, variable speeds in vivo could conceivably be linked to the ability of dynein to use a range of step sizes [53], [54], [55], to the presence of multiple ATP binding sites in each DHC [56], [57], or to the binding of regulators such as Lis1, NudE/Nde1 and Ndel1 [14], [39], [58]. Alternatively, changes in rates could reflect alterations in the resistance to movement experienced by the endosome at different points within the cell, or perhaps to switches between microtubules.

Is it important for moving endosomes to engage multiple dyneins? While in vitro experiments have shown that track and constant speed segment length increase dramatically as multiple dyneins are engaged by cargo [38], recent in vivo data reveal that reducing motor number on lipid droplets actually leads to slight increases in travel distance and speed [48], [49]. We found that the average length of constant speed segments was reduced after DHC1 depletion (Table S2), and in HeLaM cells, in which the microtubule network is less radial, the average speed of tracks >2 µm long was also reduced (Figure 5C). These results are broadly similar to changes in axonal vesicle movement following a 66% reduction in DHC1 levels [59]. Modelling studies taking account of cytoplasmic viscosity propose that cargo with one motor will be significantly more affected by increased resistance than those with two or more [60]. Hence, multiple dyneins may help native cargo maintain a fixed speed in vivo. This may be particularly important where microtubule crossovers occur, or where the microtubule encounters the actin network, as has been suggested by in vitro studies [61], [62]. Additionally, since purified dynein has a lower stalling force than kinesin [52], [53], [63], several dyneins may be necessary to counteract the action of kinesin during “tug-of-war” directional switching [37], [52]. Although switches in the direction of endosome movement were infrequent in our study, endosomes have been shown to undergo bidirectional transport [24]. However, while multiple dynein motors may be important for this in some systems [52], a single dynein motor will suffice to switch endosome direction in *Ustilago maydis* [28]. Direction switching has been proposed to need both motors to be active, with inhibition of one motor leading to loss of both directions of movement (e.g. [59], [64], [65], [66]). Our data do not fit easily with this model, since p50 expression had no effect on outward motility in HeLaM cells, even though inward movement was almost completely inhibited (Figure 5A). One explanation for this, by extension from recent work on axonal vesicle transport [59], is that the inactive dynein remaining on endosomes following p50 expression (Figure 5B) may be sufficient to generate an active plus-end-directed motor. Alternatively, our data may simply reflect the fact that most of the Rab5 endosomes we observed move only unidirectionally, perhaps because they generally possess only dynein, or more rarely only kinesin. Certainly, a 50-fold reduction in cellular dynein levels does not dramatically alter the behaviour of individual motile endosomes, despite substantially reducing the probability that endosomes will move at all.

## Materials and Methods

### Reagents

Human Rab5 was PCR amplified from a HeLa cDNA library and cloned into eGFP-C1 (Clontech) or mApple. Chicken p50, from a construct provided by Trina Schroer, Johns Hopkins University, was cloned into pcDNA3.1 containing an N-terminal mCherry tag. CC1, from a construct provided by Trina Schroer, was cloned into pcDNA3.1 containing an N-terminal RFP epitope tag. mCherry-GRASP65 was a gift from Jon Lane, University of Bristol. EMTB–3×GFP cloned into pCS2+ was a gift from Sarah Woolner, University of Manchester [42]. EB3-GFP was a gift from Anne Straube (University of Warwick). Anti-EEA1 and anti-Rab5 were from BD Transduction Labs, anti-Hrs from Alexis, anti-DYNC1HC1 from Sigma (Prestige Antibodies grade), anti-DIC (IC74.1) from Millipore, anti-TfR from Zymed, TAT1 anti-tubulin from Keith Gull, University of Oxford, and anti-LAMP1 from the Developmental Studies Hybridoma Bank, University of Iowa. Anti-Alix has been described previously [67]. Fluorescent secondary antibodies were from Jackson ImmunoResearch Laboratories. Alexa^555^-conjugated EGF was from Invitrogen.

### Cell culture and Transfection

HeLaM (provided by Andrew Peden, University of Cambridge) and hTERT-RPE-1 cells (ATCC) were grown in DMEM containing 10% FBS. HeLaM cells were transfected using JetPEI (Qbiogene). RPE cells were transfected using FuGene6 (Roche). For experiments using GFP-Rab5 alone, each 35 mm dish of cells was transfected with a mixture of 10 ng EGFP-Rab5 and 2.99 µg pBlueScript SKII as carrier. Control experiments showed that the motility of GFP-Rab5 endosomes did not alter when EGFP-Rab5 was transfected using up to 100 ng per dish. For experiments using combinations of transfected reagents, the amount of EGFP-Rab5 DNA was optimized to generate levels of GFP-Rab5 expression close to those in cells expressing 10 ng EGFP-Rab5 only. The following amounts were used: 100 ng EGFP-Rab5 plus 2.9 µg mCherry chicken p50; 50 ng EGFP-Rab5 plus 50 ng EMTB–3×GFP plus 2.9 µg pBlueScript SKII. In either single or double transfections, cells were imaged ∼24 hours after transfection. For RNAi, cells were transfected using Interferin (Qbiogene). The heavy chain of cytoplasmic dynein 1 (DYNC1HC1) was depleted over 72 hrs using a combination of the following oligonucleotides [2], [8], each at a final concentration of 6.7 nM: 5′-ACAUCAACAUAGACAUUCA-3′; 5′-GAGAGGAGGUUAUGUUUAA-3′; 5′-GCAAGAAUGUCGCUAAAUU-3′. The heavy chain of cytoplasmic dynein chain 2 (DYNC2HC1) was depleted over 72 hrs using a combination of the following oligonucleotides [51], each at a final concentration of 10 nM: 5′-GGAAUUGAAUACUCUUCAA-3′; 5′-ACAGGCUCUUCUCUCUGAA-3′. To identify cells displaying a strong functional knock-down of D1HC1, cells were transfected after 48 hrs with 50 ng mCherry-GRASP65, 100 ng EGFP-Rab5 and 2.85 µg pBlueScript SKII and examined before imaging to ensure they displayed a fully scattered Golgi complex, indicative of inhibition of dynein function. A control, scrambled RNAi (non-targeting siRNA no. 1; Perbio) was also used.

### Fractionation experiments

HeLaM cells were grown in 2×15 cm diameter dishes and transfected with mCherry alone, or mCherry-p50, using Lipofectamine 2000, for ∼16 hr. The transfection efficiency was >80%, as estimated by fluorescence microscopy. Cells were fractionated as described previously [68], generating cytosol and total membrane fractions.

### Western blotting

Western blotting was performed using HRP-conjugated secondary antibodies and ECL reagents (Perbio). Quantitative blotting to determine the efficiency of DHC1 depletion was performed using IRDye secondary antibodies and a LI-COR Odyssey scanner (LI-COR Biosciences).

### Imaging

For single color live cell imaging, HeLaM cells were grown in 35 mm glass bottomed dishes (MatTek Corporation) and imaged in HAM's F12 medium +10% FCS at 37°C using a 100X, 1.35 N.A. Phase objective and an additional 1.9X lens element, on an Olympus IX81 microscope fitted with a Optoscan high speed dynamic bandpass control monochromator (1800 g/mm Holographic grating; Cairn Research), and a Photometrics Cascade 512 back-illuminated camera (Photometrics), controlled by MetaMorph (Molecular Devices). This setup produced a scaling of 11.72 pixels/µm. After conducting preliminary experiments to establish the optimum frame rate for recording the motility of GFP-Rab5 endosomes, cells were imaged as standard for 1000 frames using continuous low-level illumination at 28 frames/s using MetaMorph in streaming mode. Control experiments showed that imaging under these conditions did not affect the probability that cells would successfully divide, or undergo apoptosis, in the succeeding 24 hours.

To assess the possible contribution of microtubule movement to fast endosome motility, cells expressing mApple-Rab5 and EMTB–3×GFP were imaged in HAM's F12 medium +10% FCS at 37°C on an Olympus IX71 microscope using a 100X 1.4 N.A. PlanApo objective with 1.6X additional magnification element. Illumination was provided by a Cairn Research LED excitation system with 470 nm and white LEDs, excitation filters (ET470/40, ET572/35) and a dichroic (T495LPXR) that allowed simultaneous excitation of the sample with blue and green light. Light was passed via a dualband GFP/mCherry dichroic to a Cairn Research TwinCam Dual Camera Emission Splitter which sent red and green light to separate Photometrics QuantEM512SC cameras using an mCherry dichroic (T585LP) and GFP (ET520/40 m) or mCherry (ET632/60 m) emission filters. The cameras were synchronised by an external trigger, and simultaneous red and green images were collected at 28 frames/s using Streampix software (Norpix), giving 9.75 pixels/µm images. TIF files from Streampix were processed in MetaMorph.

For conventional epi-fluorescence experiments, cells were grown on glass coverslips and fixed in 3% formaldehyde in PBS (or in methanol at −20°C for experiments using anti-Hrs or anti-LAMP1). EGF pulse-chase experiments were conducted as described [7]. Cells were imaged using a 60×1.4 NA Plan Apo objective on an Olympus IX70 microscope equipped for optical sectioning (Deltavision; Applied Precision). For each sample a z-series at 0.2 µm intervals was captured using a CoolSnap HQ camera (Photometrics). Images were processed using constrained iterative deconvolution, and deconvolved image stacks were projected (SoftWorx; Applied Precision). For some experiments, images were captured using an Olympus BX-60 microscope fitted with a 60×1.40 N.A. Planapo objective and a CoolSnap ES camera (Photometrics). Images were acquired using MetaVue. All images were opened as 16-bit grey-scale images and scaled using linear transformations in ImageJ, then converted to 24-bit RGB files.

### Preparation of Movies

For single color movies, the contrast of each 1000 image sequence was adjusted using the grayscale slider in MetaMorph. Regions were selected (300×200 pixels for Movie S1; 200×300 pixels for Movie S2) and a sequence of 10 seconds duration was converted to 8-bit in MetaMorph. To reduce background noise, a 2-frame rolling average was performed in ImageJ [69] using the RunningXProjector plug-in (http://valelab.ucsf.edu/~nico/IJplugins/Running_ZProjector.html), and then every other frame was deleted. Sequences from individual movies were combined into one and annotated using ImageJ. Grey-scale movies were saved in QuickTime format using the QTwriter plug-in, using MPEG-4 compression set at 65%. Play-back was set at 28 frames/s: the sequences therefore play at 2-times real-time. For the dual color ensconsin-Rab5 sequence (movie S3), a 100×100 pixel region over 150 frames in each grey-scale movie was copied and set to autoscale before making a colour overlay in MetaMorph. The movie was labelled in ImageJ and then saved as a QuickTime movie using QuickTime Pro, set at thousands of colours with no compression. Play-back is real time (28 frames s^−1^).

### Particle Tracking

For each condition, 5 representative movies were tracked. In control cells, approximately 9000 endosomes were tracked in total. Particle tracking was performed using PolyParticleTracker [29], a polynomial-fit, Gaussian-weight (PFGW) algorithm which allows accurate tracking of the extrema of intensity corresponding to individual vesicles, without errors due to a background of varying intensity or due to the presence neighbouring particles in the image. PolyParticleTracker first identifies particles to track, estimates particle coordinates, then utilizes the PFGW algorithm to achieve subpixel refinement of these coordinates. The intensity map generated for each particle by PolyParticleTracker also allows calculation of its eccentricity, radius and skewness. These values are then employed to reject intensity extrema unlikely to correspond to single vesicles. Finally, PolyParticleTracker links particle positions between frames. PolyParticleTracker, consisting of a set of Matlab scripts, is available at stacks.iop.org/PhysBio/4/220.

In order to provide an estimate of the accuracy of GFP-Rab5 tracking, tracks from a region of a movie of control cells were tracked using PolyParticleTracker and manually for comparison. Of 88 particles that could be tracked manually, all were tracked with high fidelity (i.e., without deviation from position, erroneous termination, spurious jumps) and no false-positive tracks were observed. Note that PolyParticleTracker failed to detect most fast movements when endosomes were imaged at 10 frames s^−1^, demonstrating that even robust tracking software requires very fast frame rates to capture organelle movement accurately. Static errors were estimated by analyzing tracks taken from movies of cells that were fixed and then imaged under identical experimental conditions [29], [70] (these errors are probably an overestimate, because of the effect of fixation on the intensity of GFP fluorescence and translucence of the sample). Estimates of the static error obtained by measuring the FPP distribution of the fixed sample show that non-specific fluctuations in the sample account may account for ∼10% of 0.1 µm passages, but this value declines markedly as passage length is increased.

### First passage Probability

The first passage probability distribution (*F(t,L)*) from a set of observed tracks 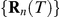 obtained from a movie or collection of movies was calculated by finding the smallest non-negative *t* that satisfies 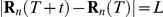, at each starting time point *T*, at each track *n* in the set. To calculate *F*(*t*,*L*), these values of *t* were counted in bins. The count was then normalized according to the total number of time points and plotted as a histogram. To express the first passage probability distribution in terms of a speed, *F*(*u*,*L*), where *u* is the particle speed, the values of *t* were replaced with *u* = *L/t* before counting and binning. Average speed distributions for each passage length were obtained using Matlab. Full details are described in [30].

### Segmentation analysis

This analysis was used to estimate the proportion of total time that endosomes spent moving in a vectorial fashion, i.e. exhibiting runs [30]. First, tracks that appeared to be dropped by the tracking routine because the particles passed momentarily out of focus were stitched together by searching for all start and end points of tracks that were close in both distance and time. Two tracks were considered to be from the same particle if they began and ended within 5 pixels and not more than 14 frames of each other.

Following this, a spatially smoothed contour was created corresponding to each particle track. This was achieved by first replacing the coordinates of each point in the particle track by the mean of all coordinates in the track which were within a threshold distance of 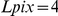 pixels (∼340 nm) to that point, then generating a coarse version of the track by successively taking sets of points in the smoothed track separated by the distance *Lpix*, starting from the initial point, and replacing each set with its mean position. This choice of *Lpix* was adequate to produce smoothed contours that closely followed the particle motion, except for extremely short range movements that were predominantly the result of imaging noise. This approach ensured that very short oscillations were not included as a *run* of active particle transport, but also carefully avoided discarding vital information, particularly that relating to the short range motions that contribute substantially to endosome motility identified using FPP analysis. Examination of individual particles in control movies showed that smoothed contours corresponding to particle tracks preserved most of these short movements. The initial and final displacements in the coarsened track were then extrapolated so that they extended further than the edge of the original track. For each contour, the position *x*(*T*) of a particle along the contour was calculated as the projection of the smoothed track along the contour, taking the projection of its initial coordinates as *x* = 0.

Using these positions, tracks were segmented into discrete runs and *rests*, using parameters that were set empirically. Positions where the particle moved less than 1 pixel (85 nm) in more than 14 frames (0.5 s) were marked as *rests*. The remaining sections are counted as *runs* if the total displacement of each was more than 1 pixel, and occurred at a rate of ≥0.17 µms^−1^.

The directionality of tracks was assessed by marking the beginning and end of tracks using Matlab and drawing a vector between these points. The angle of this vector, relative to a vector drawn between the start of the track and the centre of the cell (defined as the centre of the nucleus), was obtained. Tracks were considered as outward where this angle was <90°.

For more detailed analysis of long-range movement, tracks which attained a net displacement >2 µm were selected. Plots of displacement along the smoothed contour *vs* track duration were obtained by calculating the incremental displacement along the smoothed contour using Matlab, with each frame interval represented by a point on the curve. Plots were divided into constant-speed segments manually by scanning the plots for changes in slope. This process has been termed parsing by others [38]. To minimise noisy data, for example from fast segments attributable to recoil events or to track stitching, constant-speed segments of less than 3 frames were discounted from quantitative analysis. The speed and duration of each of the remaining segments was obtained using Matlab scripts. The position of each constant-speed segment was mapped onto the raw track trace in order to identify those that contributed to clear displacements on approximately linear trajectories. Visual inspection of plots of displacement *vs* time and raw track traces showed that these segments have speeds >0.9 µms^−1^. The statistics from such constant-speed segments were plotted as survival curves using Matlab, in which the cumulative frequency of segments exhibiting less than or equal to a particular speed is plotted.

To determine whether the movement of microtubules themselves contributes to rapid endosome translocation, twenty sequences of 1000 frames of cells expressing mApple-Rab5 and EMTB–3×GFP were acquired at simultaneously in two colours at 28 frames s^−1^, as described above. To locate Rab5 endosomes achieving fast runs, Matlab codes were used to select “inwards” tracks which attained a net displacement >2 µm and then to plot their displacement along the smoothed contour vs track duration. Continuous segments of constant speeds of >5 µms^−1^ were then manually identified and the moving endosome located in the corresponding movie. The behavior of the associated microtubule in 185 sequences was then categorized as static (60.5%); minor movement (9.8%); intermediate movement (1.6%); major movement (1%); faint microtubules that could not be scored unambiguously (15.7%); and no microtubule could be identified (11.4%).

## Supporting Information

Figure S1 **Analysis of GFP-Rab5 expression levels and DHC1 knockdown.** (A) Cells transfected with the indicated levels of GFP-Rab5 DNA were lysed and subjected to SDS PAGE and Western blot analysis using anti-Rab5, with anti-tubulin (TAT1) as a loading control. The transfection efficiency was approximately 50% for all samples. (B) HeLaM cells were transfected with control or DHC1 siRNAs, then lysed and extracts blotted for DHC1, or tubulin (TAT1) as a loading control using ECL. (C) Cells were transfected with control or DHC1 siRNAs, then lysed and assayed for protein. Protein amounts were equalised and the indicated proportions of control and DHC1 knockdown extracts were blotted for dynein intermediate chain using IC74 antibody and analysed using LI-COR Odyssey software. (D) HeLaM cells were transfected with control or DHC1 siRNAs, then homogenised and separated into crude membrane and cytosol fractions. These were blotted for DIC, or for transferrin receptor (TfR) or tubulin (TAT1) as membrane and cytosolic markers. Note that 2 x cellular equivalent of membrane was loaded compared to cytosol.
(TIF)

Figure S2 **GFP-Rab5 is a marker for early endosomes.** Cells transfected with 10 ng GFP-Rab5 DNA and 2.99 µg pBlueScript were fixed and stained for EEA1, Hrs or LAMP1 as indicated. Alternatively, cells were pulsed with Alexa^555^-conjugated EGF for 5 min and fixed. Merges are green-magenta. Scale bar = 10 µm. Insets are magnified x4.
(TIF)

Figure S3 **Tracking and FPP analysis of endosome motion in RPE cells.** GFP-Rab5 movies (1000 frames at 28 frames s^−1^) were recorded in RPE cells. (A–C) PolyParticleTracker was applied to each movie, and the resulting tracks overlaid on the first movie frame (bar = 10 µm): (A), control cells; (B), D1HC1 depleted cells; and (C), p50 expressing cells. (D–F) FPP analysis of endosome motion. FPP analysis of GFP-Rab5 in: (D), control cells; (E), D1HC1 depleted cells; and (F), p50 expressing cells. For each length of passage, *L* (see key), the probability density *F(u,L)* of that passage occurring at a given average speed is plotted.
(TIF)

Figure S4 **Processing tracks for directionality analysis.** Tracks from a representative control movie (A) were subjected to stitching. The start (white spots) and end (white crosses) of tracks with a displacement of >2 µm were highlighted (B) and this information was used to identify directionality. All traces were superimposed on the first frame of the movie. The white star corresponds to the centre of the nucleus. Bar = 10 µm.
(TIF)

Figure S5 **Rapid GFP-Rab5 endosome movement occurs along stationary microtubules.** The track of the GFP-Rab5 endosome moving along a stationary microtubule in movie S3 was smoothed and plotted as displacement versus time (red lines). Constant speed segments were colored according to the speed chart, and overlaid over the raw track (insets). The beginning (spots) and end (crosses) of each track is highlighted (inset), and the speed and duration of each segment is shown.
(TIF)

Figure S6 **Reduced dynein levels does not affect peak rates of endosome movement.** All tracks >2 µm observed under the indicated conditions were divided into constant speed segments. Survival curves were generated from these values to show rate distributions of these constant speed segments plotted as a cumulative frequency.
(TIF)

Table S1 **Inhibition of dynein markedly reduces endosome displacement.** Tracking data from HeLaM or RPE cells (5 movies analysed for each condition) were averaged to provide information about the total number of observed tracks (raw tracks), the total number of tracks after track breaks had been stitched (stitched tracks), the percentage (+/− sem) of total tracked time during which particles were undergoing *runs*, and the percentage (+/− sem) of particles that never exhibited a *run*.
(DOC)

Table S2 **DHC1 knock-down reduces the length of constant speed segments during long-range endosome motion.** GFP-Rab5 tracks obtained from 5 movies of control cells or DHC1 knock-down (kd) cells were selected for lengths >2 µm, and were divided into constant-speed segments. These were binned according to their speed (n shows the total number of segments in each bin). The average distance travelled for each bin was calculated.
(DOC)

Movie S1 Sequences of GFP-Rab5-expressing control, DHC1 knock-down and p50-expressing HeLaM cells are shown. The total duration of the original sequences is 10 seconds, with play-back set to 2-times real-time. The direction of the nucleus is indicated by the letter N. Each region is 25.6 µm wide.
(MOV)

Movie S2 Sequences of GFP-Rab5-expressing control, DHC1 knock-down and p50-expressing RPE cells are shown. The total duration of the original sequences is 10 seconds, with play-back set to 2-times real-time. The nucleus is at the bottom of each frame. Each region is 17 µm wide.
(MOV)

Movie S3 Sequence of a HeLaM cell expressing mApple-Rab5 and EMTB–3×GFP, with both channels captured simultaneously at 28 frames s^−1^ (see SI methods for details). Play-back is real time, and the image is 10 µm wide. A parsed track of the moving endosome (arrowhead) is shown in Figure S5.
(MOV)
