# Supplementary figures and images for: Roles of Dynein and Dynactin in Early Endosome Dynamics Revealed Using Automated Tracking and Global Analysis
Source: PLoS One. 2011 Sep 6;6(9):e24479. doi: 10.1371/journal.pone.0024479 (PMC3167862; doi:10.1371/journal.pone.0024479)

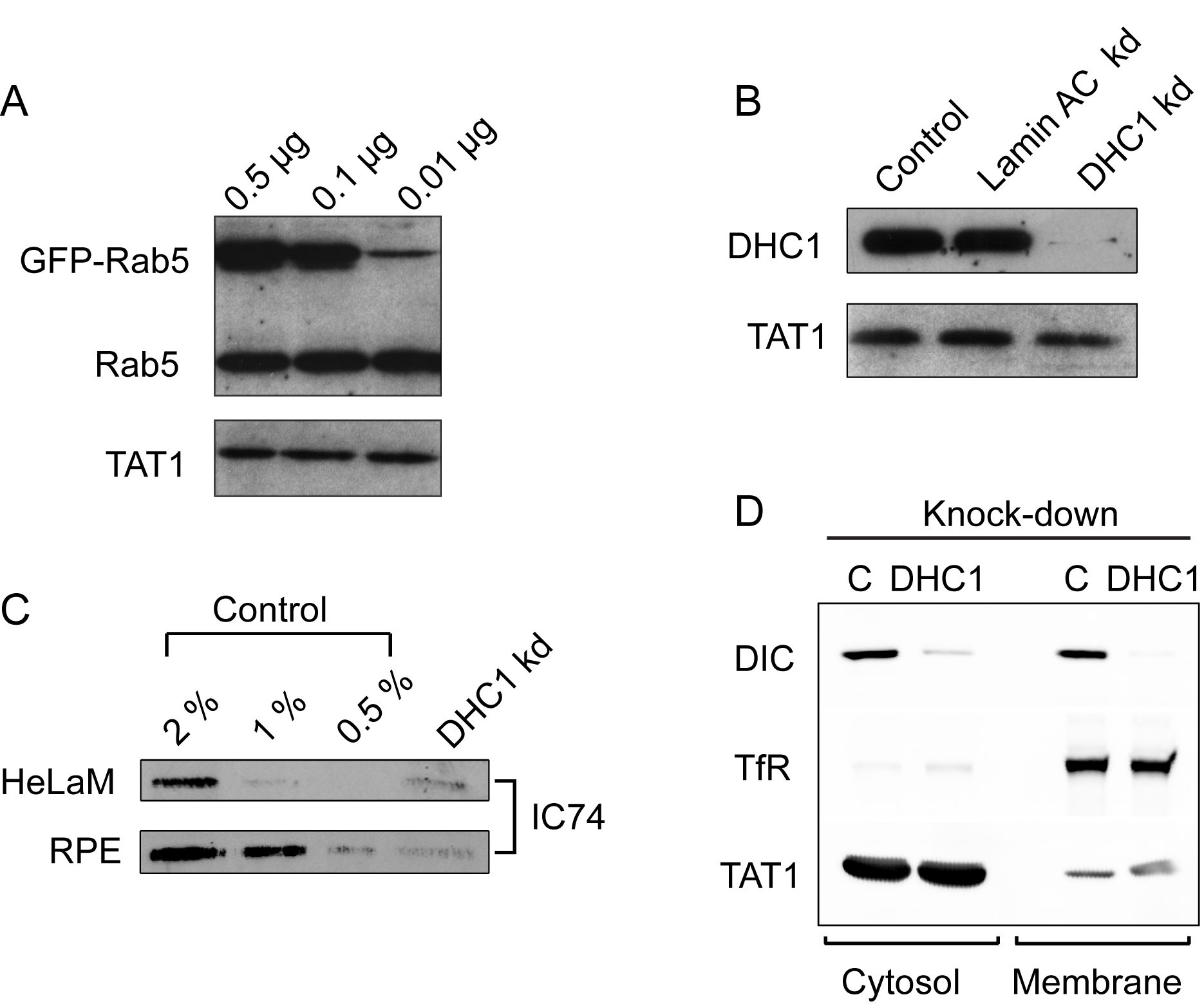

Supplement: Figure S1 — Analysis of GFP-Rab5 expression levels and DHC1 knockdown. (A) Cells transfected with the indicated levels of GFP-Rab5 DNA were lysed and subjected to SDS PAGE and Western blot analysis using anti-Rab5, with anti-tubulin (TAT1) as a loading control. The transfection efficiency was approximately 50% for all samples. (B) HeLaM cells were transfected with control or DHC1 siRNAs, then lysed and extracts blotted for DHC1, or tubulin (TAT1) as a loading control using ECL. (C) Cells were transfected with control or DHC1 siRNAs, then lysed and assayed for protein. Protein amounts were equalised and the indicated proportions of control and DHC1 knockdown extracts were blotted for dynein intermediate chain using IC74 antibody and analysed using LI-COR Odyssey software. (D) HeLaM cells were transfected with control or DHC1 siRNAs, then homogenised and separated into crude membrane and cytosol fractions. These were blotted for DIC, or for transferrin receptor (TfR) or tubulin (TAT1) as membrane and cytosolic markers. Note that 2 x cellular equivalent of membrane was loaded compared to cytosol. (TIF) [file pone.0024479.s001.tif]

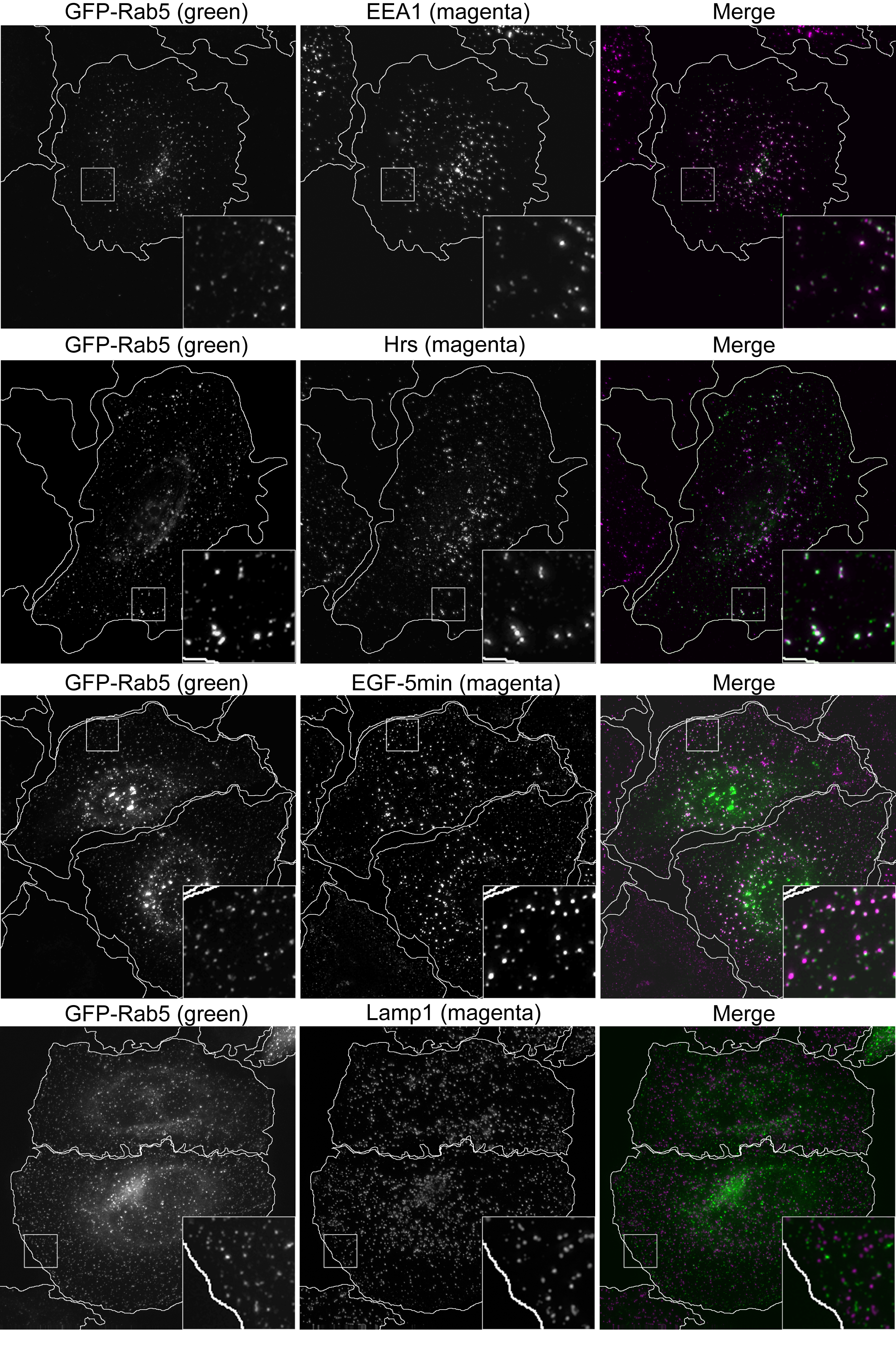

Supplement: Figure S2 — GFP-Rab5 is a marker for early endosomes. Cells transfected with 10 ng GFP-Rab5 DNA and 2.99 µg pBlueScript were fixed and stained for EEA1, Hrs or LAMP1 as indicated. Alternatively, cells were pulsed with Alexa555-conjugated EGF for 5 min and fixed. Merges are green-magenta. Scale bar = 10 µm. Insets are magnified x4. (TIF) [file pone.0024479.s002.tif]

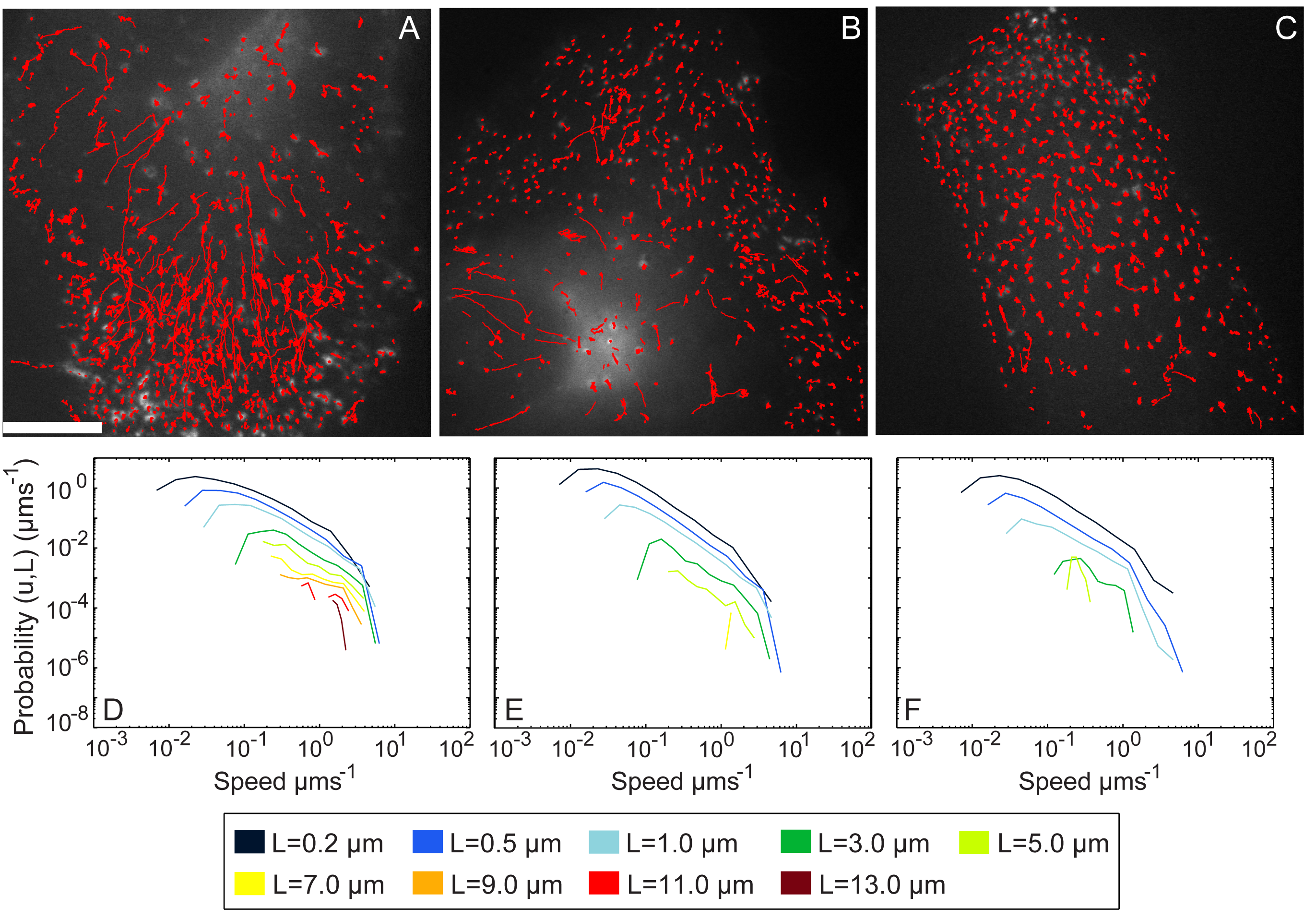

Supplement: Figure S3 — Tracking and FPP analysis of endosome motion in RPE cells. GFP-Rab5 movies (1000 frames at 28 frames s−1) were recorded in RPE cells. (A–C) PolyParticleTracker was applied to each movie, and the resulting tracks overlaid on the first movie frame (bar = 10 µm): (A), control cells; (B), D1HC1 depleted cells; and (C), p50 expressing cells. (D–F) FPP analysis of endosome motion. FPP analysis of GFP-Rab5 in: (D), control cells; (E), D1HC1 depleted cells; and (F), p50 expressing cells. For each length of passage, L (see key), the probability density F(u,L) of that passage occurring at a given average speed is plotted. (TIF) [file pone.0024479.s003.tif]

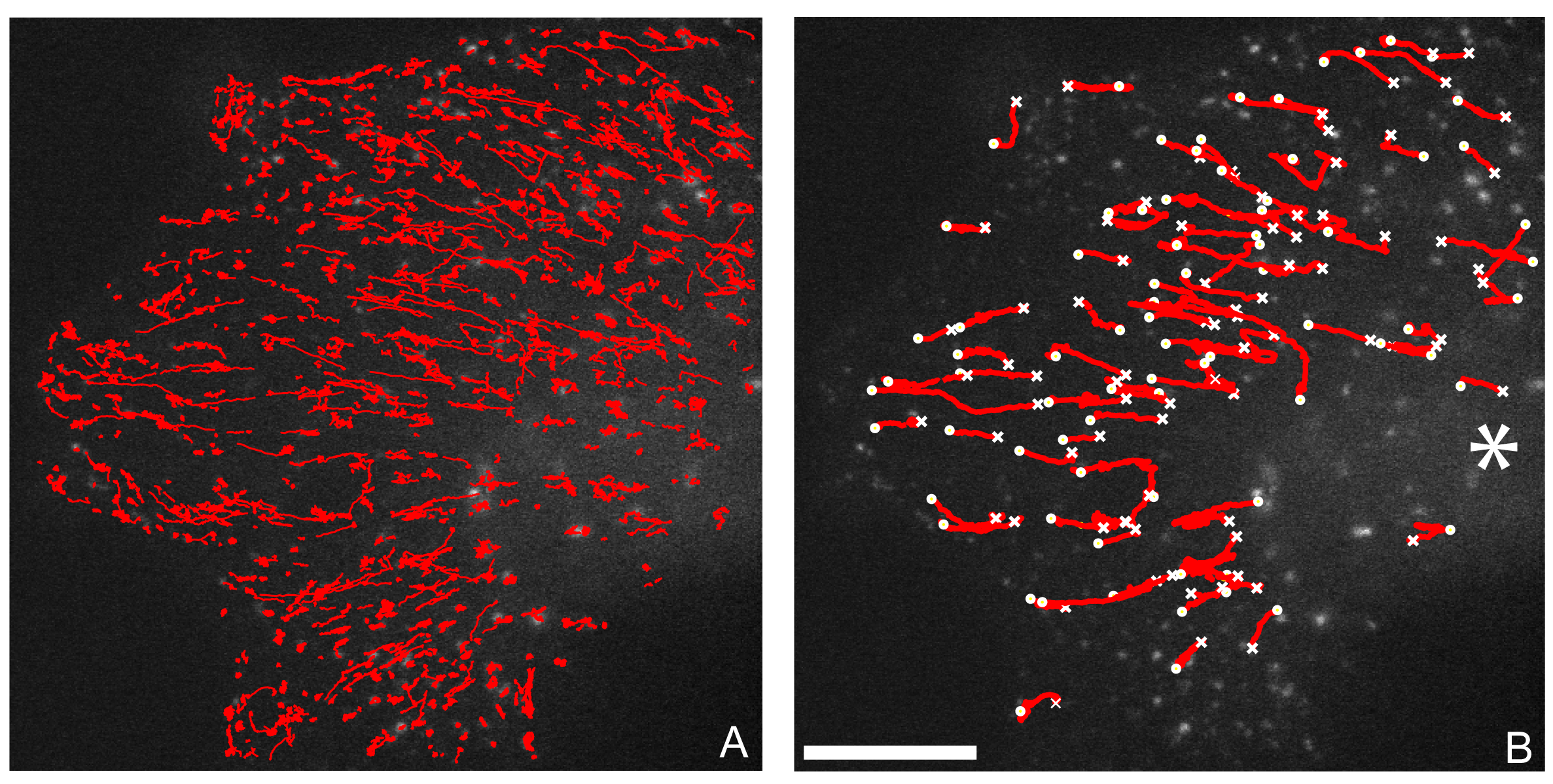

Supplement: Figure S4 — Processing tracks for directionality analysis. Tracks from a representative control movie (A) were subjected to stitching. The start (white spots) and end (white crosses) of tracks with a displacement of >2 µm were highlighted (B) and this information was used to identify directionality. All traces were superimposed on the first frame of the movie. The white star corresponds to the centre of the nucleus. Bar = 10 µm. (TIF) [file pone.0024479.s004.tif]

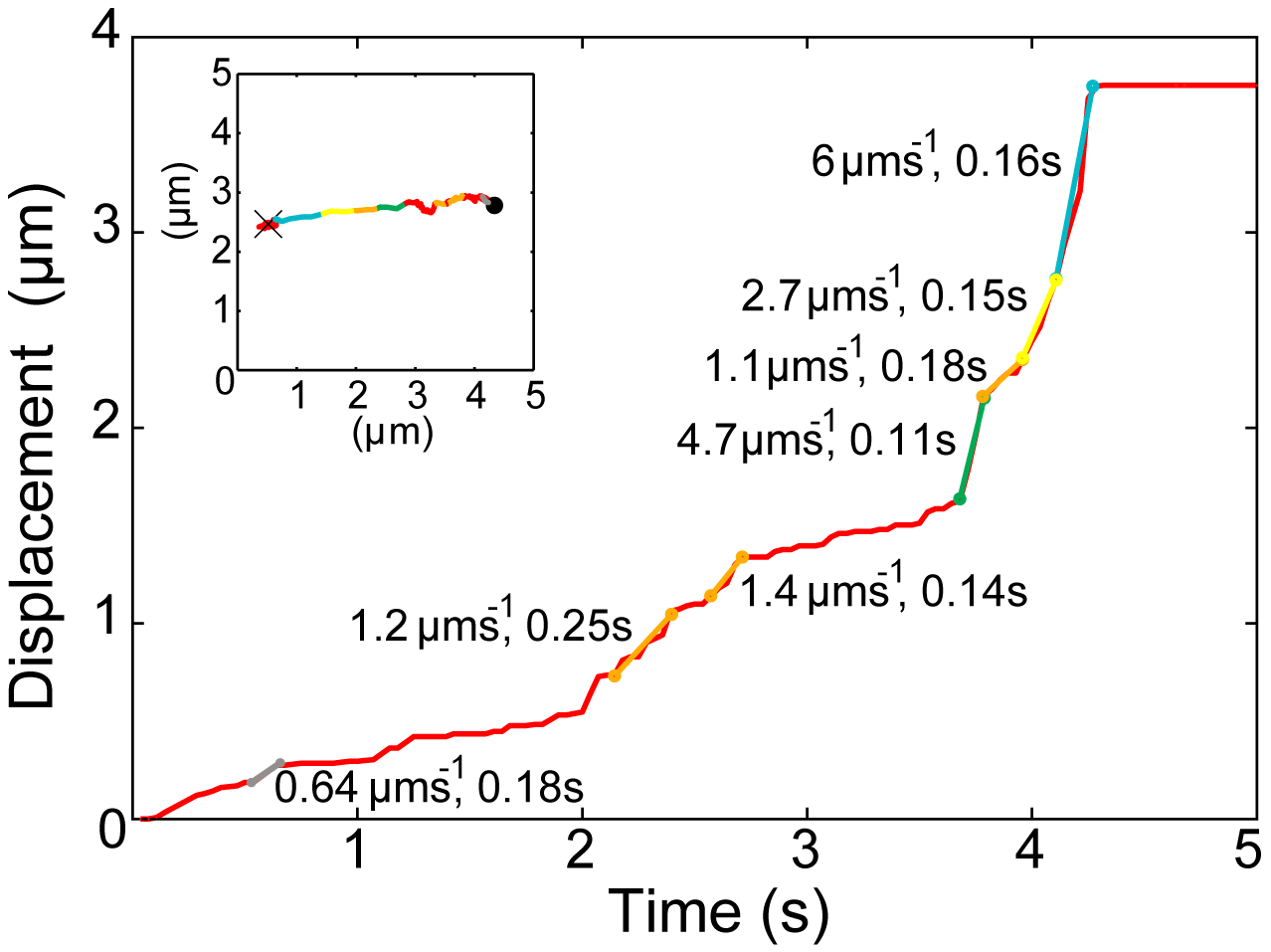

Supplement: Figure S5 — Rapid GFP-Rab5 endosome movement occurs along stationary microtubules. The track of the GFP-Rab5 endosome moving along a stationary microtubule in movie S3 was smoothed and plotted as displacement versus time (red lines). Constant speed segments were colored according to the speed chart, and overlaid over the raw track (insets). The beginning (spots) and end (crosses) of each track is highlighted (inset), and the speed and duration of each segment is shown. (TIF) [file pone.0024479.s005.tif]

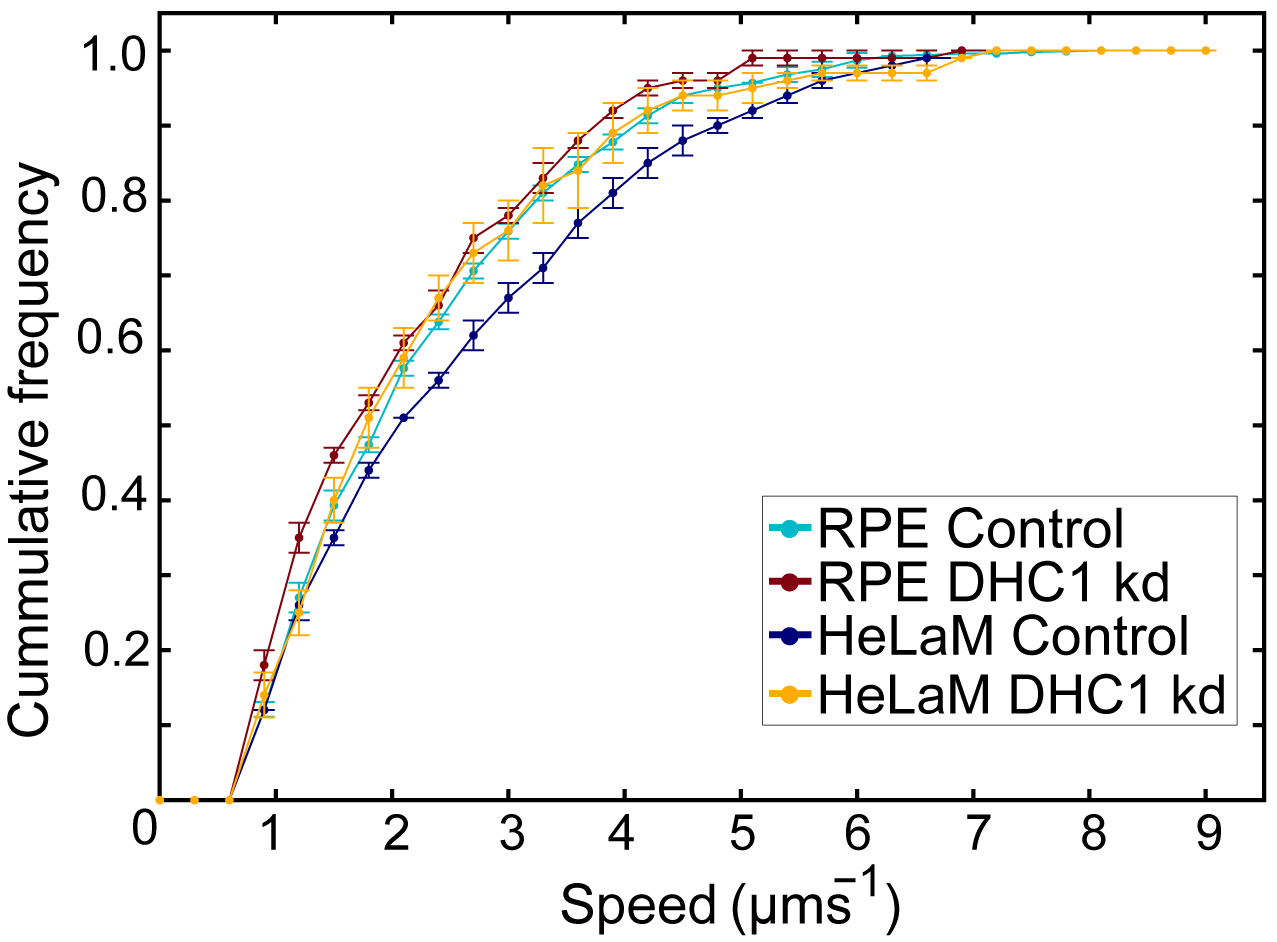

Supplement: Figure S6 — Reduced dynein levels does not affect peak rates of endosome movement. All tracks >2 µm observed under the indicated conditions were divided into constant speed segments. Survival curves were generated from these values to show rate distributions of these constant speed segments plotted as a cumulative frequency. (TIF) [file pone.0024479.s006.tif]
