# Supplementary material for: Roles of Dynein and Dynactin in Early Endosome Dynamics Revealed Using Automated Tracking and Global Analysis
Source: PLoS One. 2011 Sep 6;6(9):e24479. doi: 10.1371/journal.pone.0024479 (PMC3167862; doi:10.1371/journal.pone.0024479)
Supplement: Table S1 — Inhibition of dynein markedly reduces endosome displacement. Tracking data from HeLaM or RPE cells (5 movies analysed for each condition) were averaged to provide information about the total number of observed tracks (raw tracks), the total number of tracks after track breaks had been stitched (stitched tracks), the percentage (+/− sem) of total tracked time during which particles were undergoing runs, and the percentage (+/− sem) of particles that never exhibited a run. (DOC) [file pone.0024479.s007.doc]

|  |  | Raw tracks | Stitched tracks | Total run time (%) | Particles never move (%) |
| --- | --- | --- | --- | --- | --- |
| HeLaM | Control | 2637 | 1782 | 22 ± 2 | 35 ± 2 |
|  | DHC1 kd | 2630 | 1637 | 11 ± 1 | 54 ± 2 |
|  | p50 | 3399 | 2188 | 9 ± 1 | 60 ± 2 |
|  | CC1 | 1805 | 1101 | 10 ± 1 | 54 ± 3 |
|  | Nocodazole | 1753 | 1005 | 9 ± 1 | 57 ± 3 |
| RPE | Control | 2203 | 1379 | 9 ± 1 | 61 ± 3 |
|  | DHC1 kd | 2097 | 1317 | 3 ± 1 | 74 ± 4 |
|  | p50 | 3079 | 1720 | 4 ± 1 | 68 ± 5 |

Table S1. Inhibition of dynein markedly reduces endosome displacement.
