# Supplementary material for: Roles of Dynein and Dynactin in Early Endosome Dynamics Revealed Using Automated Tracking and Global Analysis
Source: PLoS One. 2011 Sep 6;6(9):e24479. doi: 10.1371/journal.pone.0024479 (PMC3167862; doi:10.1371/journal.pone.0024479)
Supplement: Table S2 — DHC1 knock-down reduces the length of constant speed segments during long-range endosome motion. GFP-Rab5 tracks obtained from 5 movies of control cells or DHC1 knock-down (kd) cells were selected for lengths >2 µm, and were divided into constant-speed segments. These were binned according to their speed (n shows the total number of segments in each bin). The average distance travelled for each bin was calculated. (DOC) [file pone.0024479.s008.doc]

Table S2. DHC1 knock-down reduces the length of constant speed segments during long-range endosome motion.

| Run speed range (µms^-1^) | HeLaM | | | | RPE | | | |
| --- | --- | --- | --- | --- | --- | --- | --- | --- |
|  | Control length (µm) | n | DHC1 kd length (µm) | n | Control length (µm) | n | DHC1 kd length (µm) | n |
| < 1.0 | 0.93 ± 0.14 | 42 | 1.20 ± 0.39 | 8 | 0.79 ± 0.25 | 12 | 0.56 ± 0.15 | 15 |
| 1.0 – 1.99 | 1.01 ± 0.06 | 331 | 0.87 ± 0.10 | 86 | 0.86 ± 0.04 | 452 | 0.72 ± 0.07 | 131 |
| 2.0 – 2.99 | 1.12 ± 0.08 | 180 | 0.76 ± 0.06 | 49 | 1.03 ± 0.06 | 261 | 0.87 ± 0.08 | 65 |
| 3.0 – 3.99 | 1.20 ± 0.07 | 145 | 0.90 ± 0.08 | 25 | 1.37 ± 0.11 | 156 | 0.95 ± 0.11 | 45 |
| > 4.0 | 1.10 ± 0.04 | 197 | 0.96 ± 0.07 | 29 | 1.23 ± 0.09 | 146 | 1.01 ± 0.08 | 27 |
